# A Single Case of Rosai–Dorfman Disease Marked by Pathologic Fractures, Kidney Failure, and Liver Cirrhosis Treated with Single-Agent Cladribine

**DOI:** 10.3389/fonc.2014.00297

**Published:** 2014-10-29

**Authors:** Koji Sasaki, Naveen Pemmaraju, Jason R. Westin, Wei-Lien Wang, Joseph D. Khoury, Donald A. Podoloff, Bryan Moon, Naval Daver, Gautam Borthakur

**Affiliations:** ^1^Department of Leukemia, The University of Texas MD Anderson Cancer Center, Houston, TX, USA; ^2^Department of Lymphoma and Myeloma, The University of Texas MD Anderson Cancer Center, Houston, TX, USA; ^3^Department of Pathology, The University of Texas MD Anderson Cancer Center, Houston, TX, USA; ^4^Department of Hematopathology, The University of Texas MD Anderson Cancer Center, Houston, TX, USA; ^5^Department of Nuclear Medicine, The University of Texas MD Anderson Cancer Center, Houston, TX, USA; ^6^Department of Orthopedic Oncology, The University of Texas MD Anderson Cancer Center, Houston, TX, USA

**Keywords:** Rosai–Dorfman disease, CD163, S-100, cladribine, histiocytes

## Abstract

Rosai–Dorfman disease (RDD) is a proliferative histiocytic disorder of unknown etiology, which is characterized by sinus histiocytosis with massive lymphadenopathy (1). In most cases, RDD has a benign course and treatment is not necessary. However, severe cases of RDD require treatment, and the treatment strategy is determined on the basis of the severity of the disease or the extranodal involvement of vital organs. We report a single case of RDD with atypical presentation of persistent constitutional symptoms, progressing pathologic fractures, and end-organ dysfunction, including acute kidney failure and liver cirrhosis with esophageal varices.

A 55-year-old African American woman presented with a 10-month history of night sweats, fever, weight loss, and hepatosplenomegaly. The patient had experienced an episode of transient renal failure requiring 2 weeks of dialysis 3 months before presentation. Her initial physical exam was significant for no palpable lymphadenopathy.

Positron emission tomography/computed tomography at presentation showed ^18^F fluorodeoxyglucose-avid right cardiophrenic, periportal, left gastric, and peripancreatic lymphadenopathy with a maximum node diameter of 3.5 cm and a maximum standard uptake value of 5.0; hepatosplenomegaly with craniocaudal dimensions of 22 and 14 cm for the liver and spleen, respectively; and non-displaced rib fractures at right sixth and seventh ribs.

Excisional biopsy of a right axillary lymph node revealed reactive lymph node changes characterized primarily by mantle zone and marginal zone hyperplasia with few small residual germinal centers. Multiple small non-necrotizing granulomas were identified. Sinuses were reactive and contained unremarkable sinus histiocytes without discernible emperipolesis. Immunohistochemistry showed B-cell predominance with minimal paracortical expansion.

Bone marrow biopsy demonstrated that 40–50% of cellularity with trilineage hematopoiesis. There was no morphologic evidence of infiltration by leukemia, lymphoma, or other malignancy.

Liver biopsy showed established cirrhosis, perisinusoidal fibrosis with prominent proliferation of bile ductal cells, and mild inflammation (predominantly small lymphocytes with a few plasma cells). Esophagogastroduodenoscopy revealed esophageal varices and small varices in the gastric fundus.

Eight months after presentation, the patient developed pathologic fracture of the left ischium. Positron emission tomography/computed tomography showed a lytic lesion involving the left inferior pubic ramus with a soft tissue component measuring 2.5 cm × 1.8 cm and an associated non-displaced cortical fracture of the left inferior pubic ramus at the site of the lytic lesion (Figure 1).

**Figure 1:**
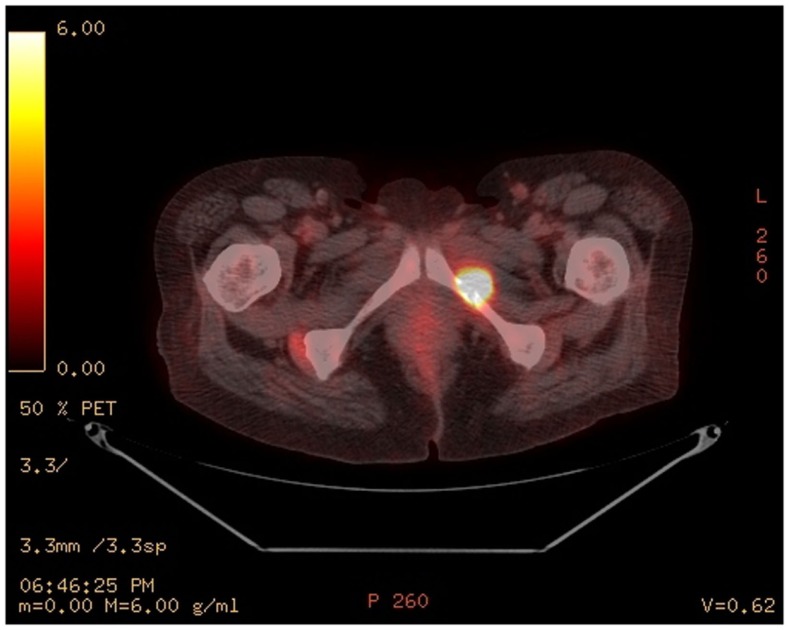
**Positron emission tomography/computed tomography of the pelvis showed a hypermetabolic lytic lesion with a pathologic fracture involving the left inferior pubic ramus with maximum SUV 15.1 associated with a soft tissue mass 2.5 cm. × 1.8 cm**.

Surgical biopsy of the left ischium revealed a mixed inflammatory infiltrate composed of large histiocytes, lymphocytes, and plasma cells. The histiocytes demonstrated emperipolesis and were reactive for both CD163 and S-100 proteins; consistent with Rosai–Dorfman disease (RDD) (Figure 2).

**Figure 2:**
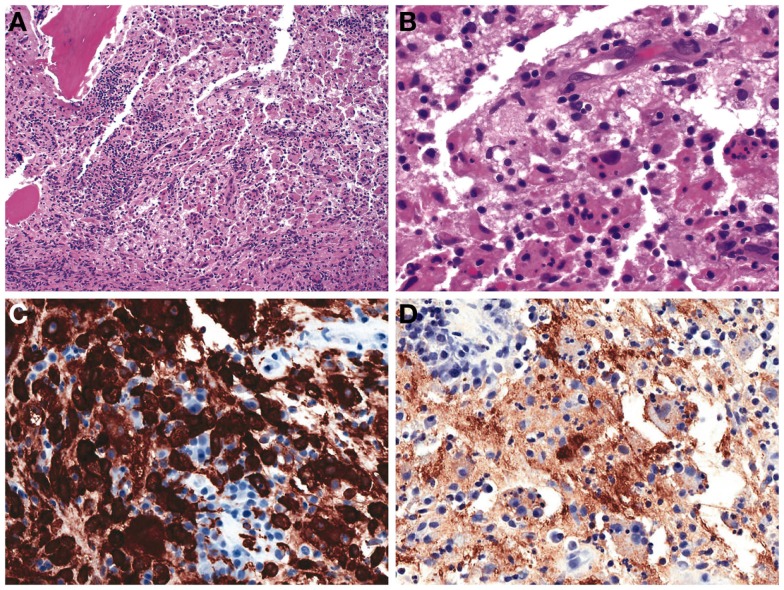
**Left ischium biopsy**. **(A)** Collections of histiocytes admixed with lymphocytes are seen involving bone (H&E, 100×). **(B)** High power reveals the histiocytes demonstrate emperipolesis, engulfing lymphocytes (H&E, 400×). Immunohistochemical studies reveal that the histiocytes co-express **(C)** CD163 and **(D)** S-100 protein.

The patient was given single-agent cladribine, 5 mg/m^2^, for days 1 through 5 in 28-day cycles. After five cycles of single-agent cladribine, magnetic resonance imaging confirmed complete resolution of the soft tissue mass in the pubic ramus and a well-healed pathologic fracture. Repeat positron emission tomography/computed tomography showed residual multicompartmental adenopathy and demonstrated no visible ^18^F fluorodeoxyglucose uptake.

In our patient, the surgical biopsy of the left ischium demonstrated histiocyte emperipolesis and CD163 and S-100 protein expression. The presence of emperipolesis by histiocytes, which are positive for S-100 and CD163, is diagnostic of RDD (2–4).

Our patient’s clinical presentation was atypical because of liver cirrhosis, kidney involvement, and confirmed pathologic fractures. The most frequent clinical presentation of RDD is massive bilateral, painless cervical lymphadenopathy with constitutional symptoms including fever, night sweats, and weight loss (5).

Skeletal lesions of RDD are typically osteolytic and can be confused radiographically with Langerhans cell histiocytosis (5). Extranodal involvement is commonly observed in the upper respiratory tract, skin, and soft tissue and less commonly observed in cases with thyroid, kidney, and skeletal involvement including spine (5–9).

^18^F fludeoxyglucose positron emission tomography can detect the metabolically highly active lesions of RDD (10). RDD often takes a self-limited benign course with frequent incidence of spontaneous resolution; nevertheless, approximately 10% of patients die of RDD due to the extranodal involvement in multiple sites and the progressive proliferation or relapse of systemic lymphadenopathy over several years (11).

In our patient, the persistent constitutional symptoms, progressive pathologic fractures, and end-organ dysfunction – including a previous episode of kidney failure requiring dialysis and liver cirrhosis with esophageal varices – justified further intervention.

Results with chemotherapeutic agents have not been encouraging, although case reports or case series have demonstrated clinical benefits or responses in patients with RDD treated with cladribine (12–15). In our patient, treatment with five cycles of cladribine resulted in radiographic improvement and stable lymphadenopathy without ^18^F fluorodeoxyglucose uptake.

In conclusion, the use of chemotherapy should be restricted to patients whose disease is life-threatening, does not respond to conservative treatments, or relapses multiple times after other treatments since RDD is self-limited in most patients. We report a single case of RDD in which treatment with single-agent cladribine resulted in symptom improvement.

## Conflict of Interest Statement

The authors declare that the research was conducted in the absence of any commercial or financial relationships that could be construed as a potential conflict of interest.
